# Leprosy in Cuba

**Published:** 1898-07

**Authors:** Wm. Thomas Corlett

**Affiliations:** M.D., L. R. C. P. Lond., Professor of Skin and Genito-Urinary Diseases in the Medical School of Western Reserve University; Physician for Diseases of the Skin to Lakeside and Charity Hospitals, Cleveland


### LEPROSY IN CUBA.

By Wm. Thomas Corlett, M.D., L. R. C. P. Lond.,

Professor of Skin and Genito-Urinary Diseases in the Medical School of Western Reserve University; Physician for Diseases of the Skin to Lakeside and Charity Hospitals, Cleveland.

Leprosy, being one of the oldest diseases with which we are acquainted, is always of general interest; but leprosy in Cuba is especially of interest at this time. It is doubtful whether the leprosy spoken of in Leviticus, which is supposed to have been written about 4,000 years ago, is identical with the leprosy as we know it to-day. Of this we are positive, that many diseases entirely distinct from leprosy were regarded as such and were spoken of in the Holy Writ. In later times the disease seems to have been widespread throughout Europe, although in England it has not existed as an epidemic disease since the 16th century. On the contrary, in Norway and Sweden, Southern France, and in Spain, the disease is still met with, and in some places it is of common occurrence. In Northern Africa, the East and West Indies, Mexico, and scattered throughout the Southern States of our own country, a few sporadic cases may still be found. Six years ago, while visiting the island of Cuba, I was especially fortunate in meeting a medical gentleman, Dr. Enrique Robelin, who is thoroughly familiar with the disease as it exists in this island. He was of the opinion that fully 200 cases existed in the city of Havana, and the disease was equally prevalent throughout the island. Lepers may be frequently seen on the streets of Havana, and in the shops mild forms of the disease may be occasionally seen. Especial effort was made to ascertain whether or not any precautions were taken to exclude them from the tobacco factories. The foreman in one of the cigar departments told me there were no lepers in his room, nor could any evidence be found to the contrary from a casual inspection of the parts of the skin exposed to view. More danger would arise from the paper used in making cigarettes, although no cases have come under my observation where the disease was presumably contracted in this way.

Especially interesting was the Hospital San Lazaro, situated in the city of Havana and containing at the time of my visit 87 cases of leprosy presenting the various stages of the disease. This hospital is one of the most celebrated now existing for the exclusive reception of this class of patients. It was founded many years ago by a gentleman, himself a leper, who bequeathed extensive lands in the suburbs of Havana to the founding of an asylum for those similarly afflicted. As the city increased in size the lands became more valuable until, at the time of my visit, the rental derived from them was more than sufficient to meet the current expenses of the hospital. It is to be regarded more as an asylum for lepers than a place of segregation; no compulsory laws exist in Cuba for the isolation of lepers, although at all times they are admitted to the

San Lazaro and cared for until they wish to change their surroundings, or are compelled to depart to that world from which no traveler returns. No effort is made to treat the disease, and they are cared for by attendants who administer to their daily wants and do what they can to mitigate their sufferings. Should American troops invade Havana, or should the island become annexed to the United States, the problem of properly caring for the lepers would naturally arise.

Much dread is manifested in coming in contact with the disease. Its contagious nature has long been known, and until we became more familiar with its methods of propagation, unnecessary fear prevailed. Through the efforts of Hansen and others we are made familiar with the active principle, the germ of the disease, which when inoculated in another gives rise to leprosy. It is only by inoculation therefore that the disease can be taken. The disease is not highly contagious, and one having a sound skin might come in daily contact with it without danger, whereas the slightest abrasion on the surface might serve as a port of entry and receive from particles of dust containing the bacillus lepræ the seed which might develop the disease. This process is slow in its action, and numerous instances are recorded where the disease has developed years after the person has migrated to a country where leprosy is unknown. The course and stages of the disease are likewise of long duration, so that years rather than months are counted in its evolution.

Before the real cause of leprosy was known many theories were advocated to account for its distribution over the globe. One of the most persistent advocates of a dietetic cause of leprosy is Jonathan Hutchinson, of London, who still maintains that an exclusive diet of fish is the cause of the disease. Coming from one so well-known to be an authority on different medical subjects, the fish theory of leprosy has received careful consideration. It is not, however, accepted by other leprologists, and to-day the germ cause of the disease is generally accepted. Experience proves, therefore, that the disease is not a menace to life when due precaution is observed. In most of the hospitals of Europe leprosy is received in the general wards with other cases. In the Hospital Saint Louis of Paris there are frequently from four to six cases in the wards, and in London there are usually a dozen or two cases scattered throughout the city at any one time. Hamburg, too, has a large contingent of lepers, being a seaport town and brought into intimate relationship with leprous countries. In Unna's hospital, two years ago, there were about a dozen cases. The danger of contact is regarded as similar to that of syphilis, to which the disease bears many points of resemblance. After inoculation the person remains in a normal condition of health for a variable length of time, which may be months or years. Finally, however, the germs of the disease develop and produce their unmistakable train of symptoms.

There are two main varieties of leprosy which clinically appear quite distinct. The onset of the two forms is the same. There is usually a general feeling of malaise, chilliness, and stupor. Indigestion is oftentimes a prominent symptom. This may subside or alternate with periods of comparatively normal health. Soon, however, the special lesions of the disease become apparent. There is usually a reddened or erythematous condition of the skin, extending over large areas, usually occurring on the face, although no part of the body is exempt. The areas are larger, covering several square inches, than are other eruptive diseases. The redness, too, is more persistent, lasting for weeks or months. The color is characteristic, being of a reddish-brown or light mahogany color, sharply defined, unaccompanied by pain or itching. At this time the symptoms of the disease vary according as one or the other form of the affection ensues.

In the *tubercular form*, reddish nodules, which vary in size from large peas to marbles, are seen in the folds of the skin. At other times the skin of the face, notably of the forehead, seems to be thickened, and the natural folds deepened, giving a rigid or lion-like countenance to the victim. As the disease progresses these tubercles enlarge, some coalesce, and finally, after years, break down and ulcerate. New lesions appear on the extremities, notably the fingers and toes, which go through the same cycle of events. When ulceration takes place, however, one section of the finger, including the bone, becomes detached. This is not always the farthest removed or distal extremity, but a section of finger may drop out, as it were, and the distal portion become contracted to take its place, thus shortening the length of the digit. At other times the fingers or toes become distorted, ankylosed, and shriveled. As the disease progresses the various parts are eaten away until, as in many cases observed in San Lazaro, the hands and feet consist of club-shaped extremities.

The throat seldom escapes, the same process going on as above described. At first the voice is husky, later a shrill whistling sound is produced in attempting to speak, and finally the patient succumbs either from exhaustion or from some concurrent malady. This extends over many years, however, and the patient a leper from youth may live to old age.

In the second clinical type, the neurotic form, the disease attacks mainly the nerve trunks, producing anesthetic areas and marked discoloration of the skin. Unquestionably the disease which we now call vitiligo was confounded with leprosy among the ancients; the white areas of this disease, especially common among the dark races, bearing some resemblance to the discoloration of the anesthetic form of leprosy. But in the former the sensibility of the skin is not impaired. There is a complete removal of pigment and an increase of pigment granules in the skin surrounding the marble-like area. Whereas, in leprosy, the spot, although of a whiter color, is not entirely devoid of pigment, and there is not an exaggerated tint in the regions surrounding. The whitened spot is usually insensitive to sharp objects thrust into the skin; thus, a needle may be inserted through the entire thickness of the skin without its being perceived. The course of this form does not differ materially from that previously described in the tubercular variety. Recessions occasionally occur, but the disease pursues, for the most part, an uninterrupted course until in from ten to thirty or forty years death comes to the patient's relief.

*The Treatment of Leprosy.*—Doubtless many cases do, when removed from debilitating conditions such as obtain in many leprous countries, such as heat, moisture, bad hygienic surroundings, insufficient food, etc., which contribute largely to hasten the course of the disease—show marked improvement, and in some instances the disease apparently leaves. It is not, however, a permanent cure, as invariably the disease returns upon any lowering of the general vitality.

Drugs of various kinds have been vaunted from time to time as a panacea for this one of the greatest of human ills. Experience has demonstrated, however, that none of them possess infallible qualities, nor can they be regarded in any way as specifics. Chaulmoogra oil has probably enjoyed the greatest popularity in the treatment of leprosy, having been first advocated by La Page, of Calcutta. From its long use and the carefully prepared reports of various observers, we are able to-day to judge of its merits. It possesses without question qualifications in the amelioration of the disease, and under its use the lesions seem to be held in abeyance if they do not entirely disappear. The disappearance, however, is not of long duration if the medicine is discontinued, for invariably the disease finally returns.

Climatic conditions and hygienic surroundings undoubtedly are of the greatest importance in its eradication. Of the sixteen or more emigrants from Norway, known to have been lepers, who settled in the Northwestern States of our own country, especially in Minnesota, but few are now known to have the disease. Either the original settlers have died out without communicating the affection to others, or the conditions there existing have proven efficient in a curative way. We have reason to believe, were the Cuban lepers to migrate to a cooler climate, where better hygienic surroundings obtained, their condition might be greatly improved. There can be no doubt, however, that much danger would ensue to those with whom they might mingle. The only way of preventing and exterminating the disease is unquestionably by segregation. This has been found extremely difficult, and in some instances well-nigh impossible. In the Sandwich Islands efforts at segregation have not always been completely executed. The island of Molokai, set aside for their reception, has been shunned by lepers, and every method has been adopted to escape the vigilance of the authorities. Segregation has been practiced for many years in the maritime provinces of Canada, notably at Tracadie, in New Brunswick, with the result of protecting the people at large and of gradually exterminating the malady in this region.

Finally, as to the danger of commingling with lepers, the sensational reports of Father Damien, who contracted the disease while acting as a missionary in the Sandwich Islands, are familiar to all. Yet with care, and understanding the method of propagation, one may associate with lepers with but little danger. Neither is there danger in allowing lepers to remain in a community, provided they are not brought into intimate relationship with those not affected. Care to cover all denuded surfaces with flexible cellodion or other protective is imperative. Care to use no drinking vessel, pipe, or other substance handled by lepers is equally necessary to avoid the disease. The paper currency in circulation in Cuba is a source of danger, and care in handling it should not be lost sight of. Finally, it should be borne in mind that anything which tends to lower the general vitality of the body predisposes to the development of the disease.—*Cleveland Medical Journal.*

